# Gain-of-function mutant p53 in cancer progression and therapy

**DOI:** 10.1093/jmcb/mjaa040

**Published:** 2020-07-28

**Authors:** Cen Zhang, Juan Liu, Dandan Xu, Tianliang Zhang, Wenwei Hu, Zhaohui Feng

**Affiliations:** Department of Radiation Oncology, Rutgers Cancer Institute of New Jersey, Rutgers-State University of New Jersey, New Brunswick, NJ 08903, USA

**Keywords:** tumor suppressor, p53, mutation, gain-of-function, tumorigenesis, cancer therapy

## Abstract

p53 is a key tumor suppressor, and loss of p53 function is frequently a prerequisite for cancer development. The p53 gene is the most frequently mutated gene in human cancers; p53 mutations occur in >50% of all human cancers and in almost every type of human cancers. Most of p53 mutations in cancers are missense mutations, which produce the full-length mutant p53 (mutp53) protein with only one amino acid difference from wild-type p53 protein. In addition to loss of the tumor-suppressive function of wild-type p53, many mutp53 proteins acquire new oncogenic activities independently of wild-type p53 to promote cancer progression, termed gain-of-function (GOF). Mutp53 protein often accumulates to very high levels in cancer cells, which is critical for its GOF. Given the high mutation frequency of the p53 gene and the GOF activities of mutp53 in cancer, therapies targeting mutp53 have attracted great interest. Further understanding the mechanisms underlying mutp53 protein accumulation and GOF will help develop effective therapies treating human cancers containing mutp53. In this review, we summarize the recent advances in the studies on mutp53 regulation and GOF as well as therapies targeting mutp53 in human cancers.

## Introduction 

Since the discovery of p53 in 1979, extensive studies have been done on p53, which have established the key role of p53 in tumor suppression (Levine et al., 2006; Muller and Vousden, 2013; Donehower et al., 2019; Levine, 2019). Loss of wild-type p53 function through mutations of the p53 gene and other mechanisms such as overexpression of negative regulators of p53 (e.g. MDM2, MDM4, and PPM1D) has been known as a prerequisite for initiation and/or progression of many human cancers (Levine et al., 2006; Muller and Vousden, 2013; Donehower et al., 2019; Levine, 2019). As a transcription factor, p53 executes its tumor-suppressive function mainly through binding to p53 DNA-binding elements in its target genes to regulate their expression. Through transcriptionally regulating these genes, p53 plays critical roles in many important biological processes, including apoptosis, cell cycle arrest, senescence, DNA repair, cell metabolism, and antioxidant defense, which contribute to p53’s function in tumor suppression (Levine et al., 2006; Muller and Vousden, 2013; Levine, 2019). The p53 gene is the most frequently mutated gene in human cancers; p53 mutations occur in >50% of all cancers. Interestingly, unlike many other tumor suppressor genes, such as BRCA1, RB, and APC, that are usually inactivated by deletions or truncating mutations in cancers, majority of p53 mutations in cancers are missense mutations, which leads to the production of full-length mutp53 proteins with only one amino acid substitution (Freed-Pastor and Prives, 2012; Muller and Vousden, 2013; Yue et al., 2017b; Mantovani et al., 2019). While p53 mutations are distributed in all coding exons of the p53 gene, the majority occur in the DNA-binding domain of p53, which impairs the ability of p53 to bind to the p53 DNA-binding elements in its target genes and thus the transcriptional activity of p53. Notably, ∼30% of p53 mutations occur at the six mutational hotspots in its DNA-binding domain, including R175, R245, R248, R249, R273, and R282 (Freed-Pastor and Prives, 2012; Muller and Vousden, 2013; Donehower et al., 2019). 

**Figure 1:**
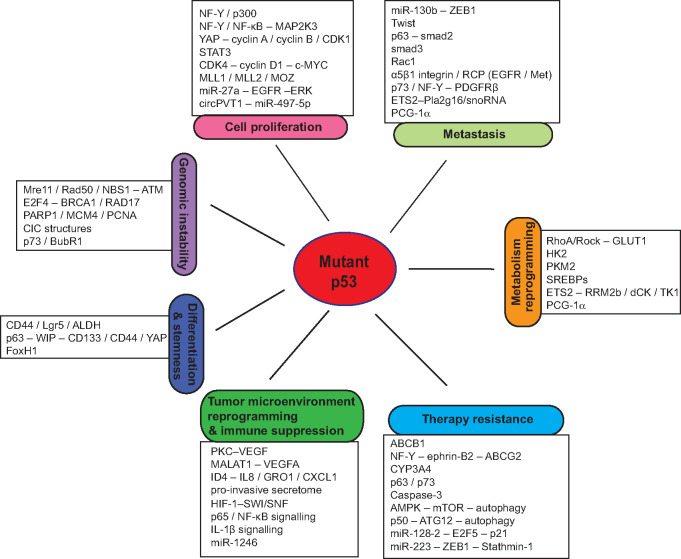
Mutp53 GOF in cancer. Mutp53 regulates cell proliferation, metastasis, genomic instability, differentiation and stemness, metabolic reprogramming, tumor microenvironment, immune response, and cancer therapy resistance to exert its GOF in tumorigenesis.

In addition to the loss of wild-type p53 function in tumor suppression, mutp53 often promotes tumor progression through the gain-of-function (GOF) mechanism. The GOF activity of mutp53 was first demonstrated in 1993, when Dittmer et al. (1993) reported that ectopic expression of R175H or R273H mutp53 endowed p53-null cells with an increased ability to form colonies in soft agar and form xenograft tumors in nude mice. Since then, numerous studies, including those using cell culture systems and mouse models and clinical studies, have shown that many missense mutp53 proteins display GOF activities to promote cancer progression, which is independent of wild-type p53 (Freed-Pastor and Prives, 2012; Muller and Vousden, 2013; Yue et al., 2017b; Mantovani et al., 2019). For instance, compared with p53 knockout mice, R172H and R270H (equivalent to human R175H and R273H, respectively) mutp53 knock-in mice develop more malignant and metastatic tumors (Lang et al., 2004; Olive et al., 2004). Humanized R248Q mutp53 knock-in mice display accelerated onset of tumors and shorter survival compared with p53-null mice (Hanel et al., 2013). For Li-Fraumeni syndrome patients who carry heterozygous germline p53 mutations, patients carrying p53 missense mutations have an earlier cancer onset compared with patients carrying p53 deletion mutations (Bougeard et al., 2008). GOF mutp53 has also been reported to be associated with poor clinical outcomes in cancer patients (Wang et al., 2014; Xu et al., 2014; Cooks et al., 2018; Schulz-Heddergott et al., 2018). Various mutp53 GOF activities have been reported so far, including promoting cell proliferation, metastasis, genomic instability, metabolic reprogramming, cell stemness, tumor microenvironment reshaping, immune suppression, and resistance to therapy in cancer (Figure 1; Freed-Pastor and Prives, 2012; Muller and Vousden, 2014; Yue et al., 2017b; Mantovani et al., 2019).

In addition to the GOF mechanism, mutp53 has also been reported to inhibit wild-type p53 function through a dominant-negative mechanism in a heterozygous situation, where both wild-type and mutp53 alleles exist (Blagosklonny, 2000; Rivlin et al., 2011). Mutp53 was reported to form heterodimer complexes with wild-type p53 to attenuate wild-type p53 function though conformational shifts or inhibiting the DNA-binding activity of wild-type p53 on target genes (Milner and Medcalf, 1991; Milner et al., 1991). Recently, an *in vitro* mutational scanning of p53 single amino acid mutants in human leukemia cells showed that missense mutants in the DNA-binding domain exert a dominant-negative effect in myeloid malignancies (Boettcher et al., 2019). Furthermore, analysis of clinical outcomes in patients with acute myeloid leukemia showed no evidence of GOF for p53 missense mutations, suggesting that mutp53 GOF may not play an important role in this type of cancer (Boettcher et al., 2019). Notably, a recent study analyzed p53 mutations in 10225 samples from 32 cancers from The Cancer Genome Atlas (TCGA) and reported that >91% of p53-mutant cancers exhibit loss of the second allele of p53 by mutation, chromosomal deletion, or copy-neutral loss of heterozygosity (Donehower et al., 2019). This implies that such a heterozygous state of p53 is often transient during cancer progression and there is a selective force driving the inactivation of the remaining wild-type p53 allele in cancers, and also suggests that the dominant-negative effect of mutp53 is not sufficient to completely inactivate the remaining wild-type p53 allele in majority of cancers (Rivlin et al., 2011; Freed-Pastor and Prives, 2012).

While mutp53 cannot bind to the p53 DNA-binding elements to transcriptionally regulate target genes of wild-type p53, mutp53 has been reported to exert its GOF activities through different mechanisms to promote tumorigenesis (Figure 1; Freed-Pastor and Prives, 2012; Muller and Vousden, 2013; Yue et al., 2017b; Mantovani et al., 2019). For instance, mutp53 binds to many different transcription factors and co-factors, such as p63, p73, NF-Y, SREBPs, Sp1, E2F1, ETS1/2, VDR, NF-κB, and regulates the transcription of their target genes (Pfister and Prives, 2017). Mutp53 also regulates expression of some genes by directly binding to specific DNA regions, such as the matrix attachment regions with a high potential for base unpairing (Will et al., 1998; Vaughan et al., 2014). Mutp53 also binds to several chromatin regulatory genes to upregulate their expression, including histone lysine methyltransferase genes MLL1 and MLL2 and histone lysine acetyltransferase gene MOZ, which in turn results in genome-wide increases of histone methylation and acetylation to regulate gene expression (Zhu et al., 2015). Furthermore, mutp53 can regulate genome-wide gene expression through directly interacting with the histone lysine methyltransferase protein MLL4 (Rahnamoun et al., 2018) or inducing chromatin remodeling via the interaction with the SWI/SNF chromatin-remodeling complex (Pfister et al., 2015) and Pontin, the AAA+ ATPase that is associated with several chromatin-remodeling complexes (e.g. Ino80, TIP60/NuA4, and SWR1 complexes) and involved in chromatin remodeling (Zhao et al., 2015). Mutp53 interacts with many different proteins other than transcription factors, including tumor suppressors and oncogenic proteins, to affect their functions. Additionally, mutp53 regulates expression of many noncoding RNAs, including microRNAs (miRNAs), circular RNAs (circRNAs), and long noncoding RNAs (lncRNA), to exert its GOF activities (Liu et al., 2017a; Di Agostino, 2020). In this review, we summarize recent advances in studies on mutp53 GOF in cancer and mutp53-targeted cancer therapies.

## Mutp53 GOF activities and mechanisms

### Cell proliferation

p53 plays a critical role in suppression of cancer cell proliferation through different mechanisms, such as cell cycle arrest, senescence, and apoptosis (Levine et al., 2006; Muller and Vousden, 2013; Levine, 2019). In contrast, GOF mutp53 promotes cancer cell proliferation. Mutp53 forms a complex with the transcription factor NF-Y and co-factor p300 and transcriptionally activates NF-Y target genes, such as cyclin A, cyclin B1, CDK1, and CDC25C, to promote cell cycle progression (Di Agostino et al., 2006). Mutp53 binds to the promoter of MAP2K3, an upstream activator of the p38 MAPK, and recruits NF-Y and NF-κB to the MAP2K3 promoter, inducing MAP2K3 expression to promote cell proliferation (Gurtner et al., 2010). Mutp53 binds to the transcription factor YAP to induce the transcription of cyclin A, cyclin B, and CDK1 to promote cell proliferation (Di Agostino et al., 2016). Mutp53 promotes colorectal tumor growth through interacting with the transcription factor STAT3 to activate STAT3 transcription program (Schulz-Heddergott et al., 2018). In addition, R249S mutp53 interacts with Pin1 after being phosphorylated by CDK4/cyclin D1 at the S249 residue and then is imported into the nucleus to stabilize c-Myc protein, resulting in the transcriptional activation of Myc target genes to promote proliferation of hepatocellular carcinoma cells (Liao et al., 2017; Wang et al., 2019).

Mutp53 also promotes cell proliferation through regulating noncoding RNAs. For instance, R273H mutp53 suppresses miR-27a expression by binding to its promoter region, which in turn activates EGFR/ERK signaling to promote cell proliferation (Wang et al., 2013). Mutp53 induces the expression of the circular RNA circPVT1 through YAP/TEAD signaling that represses miR-497-5p expression and induces the expression of cell cycle-regulated genes to promote cell proliferation (Verduci et al., 2017).

### Metastasis

p53 plays a key role in suppression of migration, invasion, and metastasis of cancer cells (Muller et al., 2011; Powell et al., 2014; Zhang et al., 2016). In contrast, promoting cancer metastasis is a well-known GOF activity of mutp53. R172H and R273H mutp53 knock-in mice develop more metastatic tumors than p53^−/−^ mice, providing clear evidence of mutp53 in promoting tumor metastasis *in vivo* (Lang et al., 2004; Olive et al., 2004). Mutp53 has been reported to promote metastasis through different mechanisms. One important mechanism is through promoting epithelial‒mesenchymal transition (EMT). Mutp53 transcriptionally represses miR-130b to upregulate ZEB1, a key EMT-related transcription factor, to promote EMT and cancer cell invasion (Dong et al., 2013). Mutp53 also promotes EMT and metastasis by upregulating the EMT-related transcription factor Twist1 (Kogan-Sakin et al., 2011) and interacting with p53 family member p63 to form a complex with Smad2 to activate the TGF-β signaling, which is important for EMT (Adorno et al., 2009). In addition to EMT, other mechanisms include modulating cell motility and extracellular matrix. For instance, mutp53 promotes metastasis by regulating SUMOylation modification of small GTPase Rac1 to activate Rac1, which plays an important role in cell motility and cancer metastasis (Yue et al., 2017a). Mutp53 promotes tumor cell invasion and motility by enhancing the interaction between α5β1 integrin and Rab-coupling protein (RCP), an important regulator of endocytic trafficking, which in turn promotes the recycling of EGFR and the protein tyrosine kinase MET (Muller et al., 2009, 2013). Mutp53 also promotes these RCP-dependent endocytic trafficking in cancer neighboring cells by exosome secretion, leading to deposition of a highly pro-invasive extracellular matrix (Novo et al., 2018). Mutp53 sequesters p73 from forming the complex with NF-Y, activating PDGF receptor β (PDGFRβ) signaling to promote pancreatic cancer metastasis (Weissmueller et al., 2014). In addition, in the R172H mutp53 knock-in mouse model, R172H mutp53 promotes tumor metastasis through interaction with the transcription factor ETS2, inducing the expression of a cluster of small nucleolar RNAs (snoRNAs) (Pourebrahim et al., 2017) and upregulating the Pla2g16 gene encoding a phospholipase that catalyzes phosphatidic acid into lysophosphatidic acid and free fatty acid, both of which are implicated in metastasis (Xiong et al., 2014).

### Genomic instability

Genome instability is a hallmark of cancer. While p53, as a guardian of genome, plays a critical role in maintaining genomic stability, GOF mutp53 promotes genomic instability, such as chromosomal and amplification instability (Hanel and Moll, 2012). For instance, fibroblasts from Li-Fraumeni syndrome patients expressing missense p53 mutations including R175H undergo S-phase reentry after being exposed to spindle depolymerizing agents that disrupt mitotic spindles, leading to the generation of polyploid cells, whereas p53-null fibroblasts are blocked from reentry (Gualberto et al., 1998). Ectopic expression of mouse R172H (equivalent to human R175H) mutp53 in p53-null primary mouse mammary epithelial cells leads to marked centrosome amplification and an increased frequency of aberrant mitosis (Murphy et al., 2000). In the pancreatic ductal adenocarcinoma mouse model, expression of R172H mutp53 and Kras (G12D) leads to the cooperative development of invasive and metastatic carcinomas with a high degree of genomic instability manifested by nonreciprocal translocations without obvious telomere erosion (Hingorani et al., 2005).

The proper DNA damage response and DNA repair function are crucial for maintaining genomic stability in cells. Mutp53 can induce genomic instability through impairing DNA damage response and DNA repair. R248W and R273H mutp53 can bind to the nuclease Mre11 and prevent the association of the Mre11‒Rad50‒NBS1 (MRN) complex to DNA double-stranded breaks (DSBs), which in turn impairs ATM activation and DNA damage response (Song et al., 2007). Mutp53 interacts with the transcription factor E2F4 and binds to the promoter region of BRCA1 and RAD17, key proteins involved in DSB DNA repair, to repress BRCA1 and RAD17 expression and impair DNA repair (Valenti et al., 2015). Mutp53 was also reported to enhance the association of the DNA repair protein PARP1 with chromatin and increase the levels of nuclear replication proteins MCM4 and PCNA, which in turn impairs DNA repair and at the same time promotes DNA replication to cause genomic instability (Polotskaia et al., 2015). In addition, other mechanisms have also been suggested to contribute to mutp53 GOF in inducing genomic instability. The p53 family member p73 plays an important role in spindle assembly checkpoint by directly interacting with BubR1, a spindle assembly checkpoint protein crucial for proper centrosome maintenance and chromosomal stability, to enhance its ability to phosphorylate downstream checkpoint effectors (Tomasini et al., 2009). Since mutp53 can bind to p73 and inhibit its transcription activity (Gaiddon et al., 2001), mutp53 may impair BubR1 function, leading to the spindle assembly checkpoint defect and aneuploidy (Hanel and Moll, 2012). Mutp53 also promotes the formation of cell-in-cell (CIC) structures via live-cell engulfment, which interferes with the cell division of host cells to result in genomic instability (Mackay et al., 2018).

### Cell differentiation and stemness

p53 promotes differentiation and restrains proliferation of stem cells, acting as a barrier of the formation of cancer stem cells (CSCs). In contrast, mutp53 displays a GOF activity to regulate dedifferentiation processes and facilitate CSC maintenance (Shetzer et al., 2016). It was reported that bone-marrow mesenchymal stem cells in Li-Fraumeni syndrome patients are tumorigenic and can induce sarcomas (Shetzer et al., 2014). Similarly, accumulation of mutp53 in progenitor-like cells in the brain subventricular zone-associated areas leads to the initiation of glioma (Wang et al., 2009). Mutp53 enhances the expression of colorectal CSC markers (e.g. CD44, Lgr5, and ALDH) by binding to CD44, Lgr5, and ALDH1A1 promoter sequences in colorectal cancer cells (Solomon et al., 2018). Mutp53 promotes proliferation and growth capacity of CSC-like cells and increases CSC markers (CD133, CD44, and YAP/TAZ) in glioblastoma and breast cancer cells by regulating WASP-interacting protein (WIP), which in turn stabilizes YAP/TAZ (Escoll et al., 2017). Mutp53 also promotes aberrant self-renewal in leukemic cells, a phenotype that is present in hematopoietic stem and progenitor cells even prior to their transformation, by upregulating FoxH1, a transcription factor involved in the regulation of stem cell-associated genes (Loizou et al., 2019).

### Metabolic reprogramming

Metabolic reprogramming is a hallmark of cancer, which sustains the needs of energy and macromolecules for the rapid growth and proliferation of cancer cells. While p53 plays a critical role in maintaining metabolic homeostasis of normal cells, GOF mutp53 promotes metabolic reprogramming in cancer cells (Labuschagne et al., 2018; Liu et al., 2019a). The enhanced aerobic glycolysis (namely the Warburg effect) is the most well-characterized metabolic change in cancer cells. Wild-type p53 has been reported to repress the Warburg effect in cancer cells through transactivating target genes that are required for oxidative phosphorylation, such as SCO_2_ (Matoba et al., 2006), as well as genes such as TIGAR and Parkin to negatively regulate glycolysis (Bensaad et al., 2006; Zhang et al., 2011; Liu et al., 2017b). In contrast, mutp53 enhances glucose uptake and glycolysis by promoting trafficking of glucose transporter GLUT1 to the plasma membrane through activation of the small GTPase RhoA and its direct downstream kinase ROCK both in cultured cancer cells and in R172H mutp53 knock-in mice, which promotes tumorigenesis (Zhang et al., 2013). Mutp53 also promotes glycolysis through enhancing the expression of glycolytic enzyme hexokinase II (HK2) and phosphorylation of PKM2 (Mathupala et al., 1997; Dando et al., 2016). Mutp53 activates the mevalonate pathway through binding to and activating the transcription factor SREBPs, which in turn induces the expression of genes in the mevalonate pathway (Freed-Pastor et al., 2012). Mutp53 enhances nucleotide synthesis via cooperating with ETS2 to activate multiple nucleotide metabolism genes, such as RRM2b, dCK, and TK1, to promote tumorigenesis (Kollareddy et al., 2015). In addition, mutp53 binds to and activates PGC-1α, a master regulator of mitochondrial biogenesis and oxidative phosphorylation, enhancing mitochondrial function to promote cancer metastasis (Basu et al., 2018). The codon 72 polymorphism of p53 (R72 or P72) influences p53 activity and is associated with the cancer risk and longevity (Zhao et al., 2018; Barnoud et al., 2019). Interestingly, PGC-1α activation by mutp53 is impacted by the codon 72 polymorphism; cancer cells with R72 variant of mutp53 show more markedly increased PGC-1α function, mitochondrial function, and metastatic capability (Basu et al., 2018).

### Tumor microenvironment and immune response regulation

Cancer cells actively shape a permissive microenvironment for cancer progression. Growing evidence has shown that mutp53 remodels the tumor microenvironment and promotes adaptation of cancer cells to the microenvironment (Stein et al., 2019). Mutp53 affects the expression of various secreted proteins to remodel the tumor microenvironment. For instance, mutp53 activates PKC to increase VEGF expression to promote angiogenesis (Kieser et al., 1994). Mutp53 forms a complex with E2F1 and binds to the promoter of inhibitor of DNA-binding 4 (ID4) to induce ID4 expression, which in turn enhances the expression of pro-angiogenic factors IL8 and GRO-α to promote angiogenesis (Fontemaggi et al., 2009). Mutp53 binds to the lncRNA MALAT1 to promote the association of MALAT1 with chromatin and induce VEGF expression in breast cancer cells (Pruszko et al., 2017). Mutp53 induces the release of a pro-invasive secretome into the tumor microenvironment through interaction with p63 (Neilsen et al., 2011). Mutp53 facilitates premetastatic niche formation by releasing exosomes to promote integrin trafficking, which enhances deposition of a highly pro-invasive extracellular matrix (Novo et al., 2018). Furthermore, mutp53 forms a complex with hypoxia-inducible factor-1 (HIF-1) that binds to the SWI/SNF chromatin-remodeling complex and induces the expression of a selective subset of hypoxia-responsive genes. Thus, mutp53 enhances HIF-1-mediated expression of some extracellular matrix components, including type VIIa1 collagen and laminin-γ2, to promote the adaptation of cancer cells to hypoxia in the tumor microenvironment (Amelio et al., 2018). In addition, mutp53 protects cancer cells from tumor-suppressive IFN-β secreted by cancer-associated fibroblasts through SOCS1-mediated inhibition of STAT1 phosphorylation (Madar et al., 2013).

The status of p53 in cancer cells has a profound impact on the immune response, resulting in various outcomes that can impede or support cancer development (Blagih et al., 2020). It was reported that the expression of mutp53 in human lung cancer correlates with increased PD-L1 expression, which may help to identify patients responsive to checkpoint inhibitors targeting PD-L1 (Dong et al., 2017). NF-κB plays a key role in regulating immune response to chronic inflammation. Mutp53 activates NF-κB signaling by promoting p65 translocation to the nucleus or inhibiting tumor suppressor DAB2IP (Cooks et al., 2013; Di Minin et al., 2014). R273H mutp53 transcriptionally represses IL-1 receptor antagonist (IL-1RA) to sustain IL-1β signaling (Ubertini et al., 2015). In addition, R248W mutp53 increases exosome secretion of miR-1246 to reprogram macrophages to tumor-supporting macrophages (Cooks et al., 2018). Thus, through the mutp53 GOF mechanism, cancer cells can reprogram macrophages and other myeloid subsets to support cancer development.

### Cancer therapy resistance

p53 induces apoptosis, cell cycle arrest, senescence, and other biological processes to mediate cancer cell response to therapies. In contrast, GOF mutp53 has been reported to promote therapeutic resistance in cancer (He et al., 2017; Zhou et al., 2019). Enhanced drug efflux through upregulation of ATP-binding cassette (ABC) transporters that extrude drugs out of cells is an important mechanism for multidrug resistance. While p53 represses the expression of ABC transporter ABCB1, GOF mutp53 induces ABCB1 expression to mediate the ATP-dependent efflux of drugs from cells to promote chemoresistance (Chin et al., 1992). Mechanistically, mutp53 is recruited to the ABCB1 promotor through interacting with ETS1 to activate ABCB1 transcription (Sampath et al., 2001). Mutp53 interacts with NF-Y to induce the expression of ephrin-B2, a ligand for the receptor tyrosine kinases ephrin receptors, which in turn upregulates the expression of the ABC transporter ABCG2 to promote chemoresistance (Alam et al., 2016). Cytochrome P450 (CYP450) family members are key enzymes in drug metabolism, mediating the process of drug oxidation. Mutp53 (e.g. R282W) induces CYP450 enzyme 3A4 (CYP3A4) expression to promote resistance to several CYP3A4-metabolized chemotherapeutic drugs (Xu et al., 2014).

Mutp53 also promotes chemoresistance by inhibiting apoptosis and autophagy. Mutp53 binds to p63 and p73 and represses their transcriptional activities to inhibit apoptosis induced by chemotherapeutic agents (Di Como et al., 1999; Gaiddon et al., 2001). Mutp53 interacts with AMPKα to repress AMPK signaling, activating mTOR to suppress autophagy (Zhou et al., 2014). Furthermore, mutp53 inhibits autophagy by forming a complex with p50 of NF-κB and binding to the promoter of autophagic gene ATG12 to repress its expression (Cordani et al., 2016).

Mutp53 also regulates miRNA expression to promote chemoresistance. For instance, R175H mutp53 induces the expression of miR128-2, which targets the transcription factor E2F5 to upregulate p21, to inhibit apoptosis and confers resistance to cisplatin, doxorubicin, and 5-fluorouracyl treatments (Donzelli et al., 2012). Furthermore, mutp53 (e.g. R175H) downregulates miR-223 expression in cancer cells to induce chemoresistance through binding to miR-223 promoter to reduce its expression via the transcriptional repressor ZEB-1, which in turn induces Stathmin-1, an oncoprotein that confers chemoresistance partially through regulating microtubule dynamics (Masciarelli et al., 2014).

## Mutp53 protein accumulation and regulation

**Figure 2:**
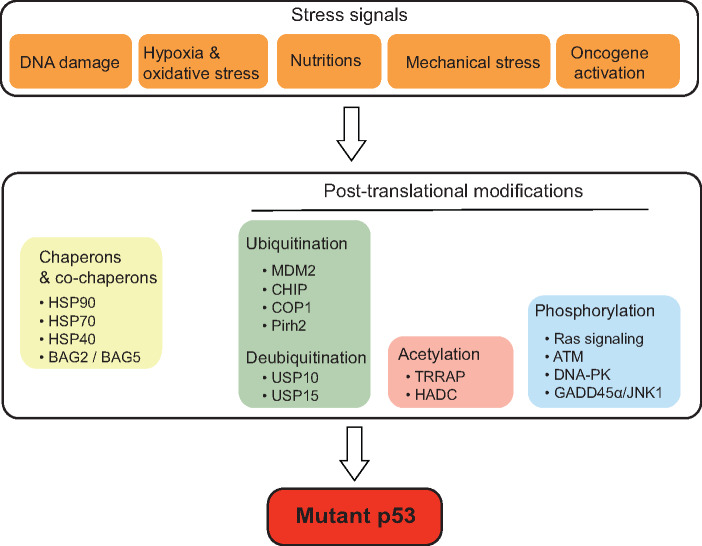
The regulation of mutp53 protein in cancer. Mutp53 protein accumulates to very high levels in cancer cells. Mutp53 protein levels in cancer cells are regulated by different mechanisms, including posttranslational modifications (such as ubiquitination, acetylation, and phosphorylation), chaperones and co-chaperone proteins, as well as different stress signals.

p53 protein is exquisitely regulated by many different mechanisms to maintain its proper levels and function in cells. Among these mechanisms, posttranslational modifications represent a very efficient and critical one for p53 regulation. The posttranslational modifications include ubiquitination, phosphorylation, acetylation, methylation, sumoylation, etc., which affect p53 protein stability, conformation, localization, and interaction with other proteins (Levine, 2019; Liu et al., 2019b). The E3 ubiquitin ligase MDM2, which directly binds to p53 and ubiquitinates it for proteasomal degradation, is the most critical negative regulator of p53 in cells. Meanwhile, MDM2 is a direct target of p53; p53 transcriptionally induces MDM2. Thus, MDM2 and p53 form a negative feedback loop to tightly regulate p53 protein levels (Zhao et al., 2014). Mutp53 protein is frequently stabilized and accumulated to very high levels in tumor tissues, which is required for the execution of its GOF activities (Freed-Pastor and Prives, 2012; Yue et al., 2017b). Currently, the mechanism of mutp53 accumulation in cancer is incompletely understood. Recent studies have shown that mutp53 can be regulated by posttranslational modifications (e.g. ubiquitination, acetylation, phosphorylation, etc.), chaperones and co-chaperone proteins, and different stress signals (Figure 2).

The inability of mutp53 to transcriptionally induce MDM2 was considered to account for mutp53 accumulation in tumor tissues (Freed-Pastor and Prives, 2012; Muller and Vousden, 2014). However, studies in mutp53 knock-in mouse models show that mutp53 is only accumulated in tumors but not normal tissues, and knockdown of MDM2 promotes mutp53 accumulation in both normal tissues and tumors, suggesting that mutp53 is inherently unstable and can be degraded by MDM2 *in vivo* like wild-type p53 in normal tissues and that some changes in tumors inhibit mutp53 degradation by MDM2 (Lang et al., 2004; Olive et al., 2004). Notably, studies also show that MDM2 can ubiquitinate and degrade mutp53 in cultured cancer cells (Lukashchuk and Vousden, 2007; Terzian et al., 2008; Zheng et al., 2013). In addition to MDM2, other E3 ubiquitin ligases for p53, such as CHIP, COP1, and Pirh2, can also ubiquitinate mutp53 and promote its degradation (Lukashchuk and Vousden, 2007; Yan et al., 2014). MDM2 isoform B is a splice variant of MDM2 that lacks the p53-binding domain but retains the ability to interact with full-length MDM2. MDM2 isoform B is frequently overexpressed in cancers and inhibits mutp53 ubiquitination and degradation through blocking full-length MDM2, which in turn promotes mutp53 accumulation in cancer cells (Zheng et al., 2013).

Chaperones, such as the heat-shock proteins (HSPs), interact with newly synthesized proteins to promote their proper folding and help refold damaged or misfolded proteins. HSP90 forms a complex with mutp53 to inhibit ubiquitination and degradation of mutp53 by MDM2 and CHIP (Peng et al., 2001; Li et al., 2011b). Histone deacetylase 6 (HDAC6) activates HSP90 activity to inhibit MDM2- and CHIP-mediated mutp53 ubiquitination and degradation (Li et al., 2011a). Interestingly, HSP70 not only inhibits MDM2-mediated ubiquitination and degradation of mutp53 but also promotes the formation of amyloid-like aggregation for mutp53 R175H, both of which promote mutp53 stabilization (Wiech et al., 2012). DNAJA1, a member of HSP40 family, stabilizes mutp53 by competitively binding to CHIP ubiquitin ligase (Parrales et al., 2016). In addition, BAG2 and BAG5, two co-chaperone proteins, bind to mutp53 to inhibit its degradation by MDM2 and CHIP (Yue et al., 2015, 2016).

Some deubiquitinases have been reported to be involved in the regulation of mutp53. USP10 deubiquitinates both wild-type p53 and mutp53 (Yuan et al., 2010). In renal cell carcinoma (RCC), USP10 is overexpressed only in tumors expressing mutp53 but undetectable in tumors expressing wild-type p53. The overexpression of USP10 in p53-mutant RCCs inhibits MDM2-mediated mutp53 ubiquitination, leading to mutp53 accumulation (Yuan et al., 2010). USP15 inhibits the nuclear export, ubiquitination, and lysosome-mediated degradation of R175H mutp53 independently of MDM2 in ovarian cancer cells (Padmanabhan et al., 2018).

In addition to ubiquitination and deubiquitination, the acetylation modification also plays an important role in the regulation of mutp53. TRRAP, a constituent of several histone acetyltransferase complexes, induces mutp53 stabilization via blocking MDM2 function in lymphoma (Jethwa et al., 2018). Inhibition of HDACs, specifically HDAC1/2/3, induces mutp53 degradation (Jethwa et al., 2018). Interestingly, HDAC1/2 also directly binds to the promoter region of mutp53 gene to increase the mutp53 mRNA expression in pancreatic ductal adenocarcinoma cells (Stojanovic et al., 2017).

The phosphorylation modification also regulates mutp53 protein levels and/or functions. Interestingly, different phosphorylation modifications show different effects upon mutp53, and the mechanism is not well understood. For instance, some phosphorylation modifications, such as S6/S9 phosphorylation of mutp53 R280K by Ras signaling, S15 phosphorylation of mutp53 R248W and R273H by ATM, and S15/S37 phosphorylation of mutp53 R175H and R273H by DNA-PK, were reported to stabilize mutp53 and enhance its GOF activities (Song et al., 2007; Adorno et al., 2009; Sonego et al., 2013). In contrast, some other phosphorylation modifications, such as S15 phosphorylation of mutp53 P223L and V274V via JNK1 activation, were reported to restore the wild-type p53 function of mutp53 (Zerbini et al., 2005).

It has been well established that wild-type p53 can be stabilized and activated by a wide variety of stress signals, including DNA damage, hypoxia, nutritional deprivation, and activation of oncogenes (Levine et al., 2006, 2019; Muller and Vousden, 2014). Interestingly, recent studies showed that mutp53 can also be regulated by different stress signals, including oxidative stress, proteotoxic stress, mechanical constraints, nutrient limitations, hyperproliferation-related DNA damage, and hypoxia, contributing to stabilization, accumulation, and GOF of mutp53 (Mantovani et al., 2019). For example, DNA damage, oxidative stress, and activation of oncogenes (e.g. Myc and Ras) induce mutp53 accumulation (Suh et al., 2011). Oxidative and proteotoxic stress can induce stabilization of heat-shock factor 1 (HSF1), which stimulates HSP90 transcription to stabilize mutp53. In turn, mutp53 interacts with HSF1 to facilitate HSF1 recruitment to the promoter region of HSP90 to induce HSP90 expression, which forms a feed-forward loop to sustain mutp53 accumulation (Li et al., 2014). Constitutive activation of DNA damage checkpoint signaling promotes mutp53 stabilization through ATM-mediated regulation of ubiquitin/proteasome activity toward mutp53 (Frum et al., 2016). Cancer-associated fibrosis generates a dense and mechanically rigid extracellular matrix as an extracellular mechanical signal, leading to RhoA geranylgeranylation and HDAC6/Hsp90-dependent mutp53 stabilization in cancer cells (Ingallina et al., 2018). Nutrient availability also affects mutp53 stabilization and accumulation in tumors. For instance, mevalonate pathway activation has been reported to promote mutp53 stabilization and accumulation in cells (Parrales et al., 2016; Ingallina et al., 2018). In contrast, glucose restriction induces mutp53 degradation through chaperone-mediated autophagy (Rodriguez et al., 2012). Taken together, these results suggest that stress signals modulate mutp53 accumulation and GOF through its posttranslational modification, adding a new layer of regulation for mutp53 in cancer cells.

## Therapeutic strategies to target mutp53

**Figure 3:**
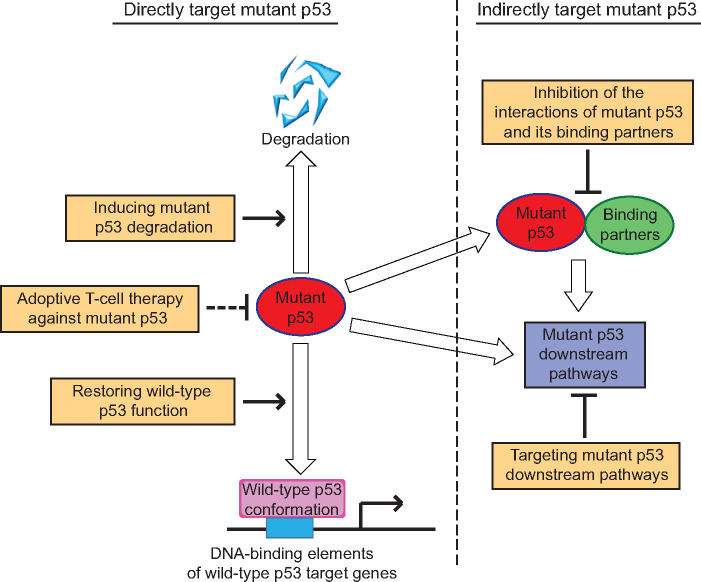
Therapeutic strategies targeting mutp53 in cancer. The therapeutic strategies targeting mutp53 in cancer include targeting mutp53 directly or indirectly. The direct strategies include restoring wild-type p53 function to mutp53, inducing mutp53 degradation, and adoptive T-cell therapy against mutp53. The indirect strategies include inhibition of the interactions between mutp53 and its binding partners and targeting critical downstream pathways of mutp53.

Given that the p53 gene is mutated in >50% of all human cancers and mutp53 frequently displays GOF activities, mutp53 has become an attractive target for cancer therapy. Based on the facts that mutp53 is frequently accumulated to very higher levels in tumor tissues, loses transcriptional activity of wild-type p53, and frequently acquires GOF activities through interacting with other proteins and/or regulating critical downstream signaling pathways, different strategies have been developed to target mutp53 for cancer therapy. These therapeutic strategies can be classified into two major categories (Figure 3). The first is to target mutp53 directly by restoration of the wild-type tumor-suppressive function of p53 or deprivation of mutp53 through inducing its degradation. The second is to target specific mutp53-binding proteins or critical downstream signaling pathways of mutp53 to inhibit mutp53 GOF activities (Blandino and Di Agostino, 2018; Ladds and Lain, 2019; Zhou et al., 2019).

### Restoring wild-type p53 function

Since majority of p53 mutations in cancers are missense mutations, the idea to convert mutp53 to the wild-type p53 conformation and restore its transcriptional activity has attracted many studies. CP-31398, a styrylquinazoline compound, was identified to be able to restore the wild-type p53 conformation and transcriptional activity in cancer cells expressing mutp53 and inhibit their proliferation in 1999 from a high-throughput screen (Foster et al., 1999). Identified from screening a small library of compounds by *in vitro* DNA-binding assays, p53R3 can inhibit the proliferation of cancer cells expressing mutp53 by inducing the expression of p53 target genes, including p21, PUMA, and BAX, to induce cell cycle arrest and apoptosis of cancer cells (Weinmann et al., 2008). Identified from a cell-based luciferase-reporter screen, Chetomin specifically reactivates R175H mutp53 to the wild-type p53 conformation through increasing its binding capacity with DNAJB1 (Hsp40), which selectively inhibits the growth of cancer cells with R175H but not R273H mutp53 (Hiraki et al., 2015). It is known that a single zinc ion binds to p53 near the DNA-binding interface, which is critical for the conformation and transcriptional activity of wild-type p53, and zinc treatment can restore the wild-type conformation and DNA-binding activity to R175H and R273H mutp53 in cancer cells (Puca et al., 2011). NSC319726 (also known as ZMC1), a zinc metallochaperones, restores wild-type conformation and function to R175H mutp53 through its zinc ion chelating activity (Yu et al., 2012). COTI-2, a recently developed zinc ion chelator, can restore wild-type conformation to mutp53 and inhibit growth of tumors expressing mutp53 (Synnott et al., 2020).

Identified by the screen for compounds that suppress the growth of cancer cells expressing mutp53, PRIMA-1 covalently binds to thiol groups of R175H, R248Q, and R273H mutp53, refolding mutp53 into a wild-type conformation (Bykov et al., 2002). Methylated analog of PRIMA-1, PRIMA-1MET (also known as APR-246), is more potent and less toxic than PRIMA-1 (Lambert et al., 2009). The natural compound phenethyl isothiocyanate from cruciferous vegetables restores the wild-type conformation and transcription activity of R175H mutp53, sensitizes the R175H mutp53 to proteasomal degradation, and shows a growth inhibitory effect on cancer cells expressing R175H mutp53 (Aggarwal et al., 2016).

Interestingly, recent studies have suggested that some GOF mutp53 (e.g. R248Q, R248W, and R175H) can form protein aggregates, contributing to mutp53 GOF activities. Suppressing the aggregation of mutp53 abrogates its GOF activities and restores activity of wild-type p53 (Silva et al., 2018). For instance, ReACp53, a cell-penetrating peptide, can inhibit mutp53 aggregation and rescue p53 function in cancer cells and organoids derived from high-grade serous ovarian carcinomas, an aggressive cancer characterized by ubiquitous p53 mutations (Soragni et al., 2016). Polyarginine, a synthetic cationic peptide, can inhibit mutp53 aggregation to suppress the proliferation of p53-mutant lung cancer H719 (R248Q) and breast cancer SK-BR-3 (R175H) cells (Chen et al., 2017).

### Inducing mutp53 degradation

Given that mutp53 is frequently accumulated to high levels in cancer cells to exert its GOF activities, inducing mutp53 degradation should be an effective strategy for cancer therapy. Since the interaction of mutp53 with the HDAC6/HSP90 chaperone complex is critical for mutp53 stabilization in cancer cells, disruption of the HDAC6/HSP90 complex by inhibitors of HSP90 or HDAC6 has been shown to be a promising strategy to induce mutp53 degradation (Peng et al., 2001; Li et al., 2011a, b). Geldanamycin is the first HSP90 inhibitor used for targeting mutp53 to induce its degradation (Blagosklonny et al., 1995). 17-AAG, an analog of Geldanamycin, induces proteasomal degradation of mutp53 through MDM2 and CHIP-mediated ubiquitination (Li et al., 2011b). Ganetespib, another HSP90 inhibitor, has a much higher potency in mutp53 degradation and has entered the phase III clinical trials (Alexandrova et al., 2015). The HDAC inhibitors, such as trichostatin A, FR901228, and SAHA, can also induce the proteasomal degradation of mutp53 mediated by MDM2 and CHIP (Blagosklonny et al., 2005; Li et al., 2011a). Activation of the mevalonate pathway and RhoA- and actin-dependent transduction of mechanical inputs, such as the stiffness of extracellular environment, have been reported to promote mutp53 stabilization and accumulation in cancer cells (Parrales et al., 2016; Ingallina et al., 2018). Inhibition of critical enzymes in the mevalonate pathway, such as inhibition of 3-hydroxy-3-methylglutaryl coenzyme A (HMG-CoA) reductase by Statins, geranylgeranyl transferase 1 (GGTase 1) by GGTI-298, and farnesyl diphosphate synthase (FDPS) by zoledronic acid, was shown to destabilize mutp53 and inhibit the growth and invasion of cancer cells expressing mutp53 (Freed-Pastor et al., 2012; Ingallina et al., 2018). In addition, Statins also induce CHIP-mediated degradation of mutp53 by blocking the interaction between mutp53 and DNAJA1 (Parrales et al., 2016). Gambogic acid, a natural product derived from the *Garcinia hanburyi* tree, reduces mutp53 levels by targeting the CHIP-mediated nuclear export and ubiquitination of mutp53 (Wang et al., 2011).

In addition to proteasomal degradation, mutp53 can also be degraded through autophagy/lysosomal degradation. For instance, spautin-1, a small molecule designed for inhibition of macroautophagy, induces the lysosomal degradation of mutp53 through the HSC70-mediated autophagy pathway to kill cancer cells expressing mutp53 (Vakifahmetoglu-Norberg et al., 2013). MCB-613, a small-molecule stimulator of steroid receptor coactivators, induces ubiquitination, nuclear export, and degradation of R175H mutp53 through a lysosome-mediated pathway by depletion of USP15, leading to catastrophic death of ovarian cancer cells (Padmanabhan et al., 2018).

### Inhibition of the interactions of mutp53 with its binding partners

Mutp53 interacts with many proteins, including transcription factors and nontranscription factors, to exert its GOF activities. Therefore, blocking these interactions has been tested as a strategy to treat cancers with mutp53. Identified from a screen of compounds that reactivate the transcriptional activity of wild-type p53 in mutp53, the small molecule RETRA can release p73 from the mutp53/p73 complex, resulting in the tumor-suppressive effect similar to the functional reactivation of p53 (Kravchenko et al., 2008). The small molecule prodigiosin induces p73 expression and disrupts p73 interaction with mutp53, thereby displaying antitumor effects (Hong et al., 2014).

### Targeting critical downstream pathways of mutp53

Mutp53 frequently displays GOF activities through regulating different downstream signaling pathways in cancer cells. Therefore, targeting some critical downstream pathways of mutp53 provides an alternative strategy for treating cancers expressing mutp53. For instance, mutp53 upregulates EGFR/integrin recycling and PDGFRβ to promote tumor metastasis, and thus inhibition of EGFR by cetuximab or inhibition of PDGFRβ by imatinib blocks cancer metastasis (Muller et al., 2009; Weissmueller et al., 2014). The Rac1 inhibitor NSC23766 inhibits mutp53 GOF activity in tumor growth and metastasis through blocking the activation of Rac1 signaling by mutp53 (Yue et al., 2017a). The ROCK inhibitor Y27632 blocks RhoA/ROCK pathway activated by mutp53 and inhibits mutp53 GOF in promoting glycolysis and tumorigenesis (Zhang et al., 2013). The AAA+ ATPase Pontin is a mutp53-specific binding protein that enhances mutp53 transcriptional activity and GOF, and blocking the ATPase activity of Pontin by rottlerin has been shown to compromise mutp53 GOF in tumor growth and metastasis (Zhao et al., 2015).

A growing body of evidence has demonstrated that mutp53 often renders cancer cells dependent on some downstream pathways for survival, and inhibition of these pathways leads to synthetic lethality, providing new therapeutic targets for tumors expressing mutp53. For example, p53 mutations result in loss of the G1/S checkpoint, leading to the dependency of cells on the G2/M check point. Therefore, inhibition of G2/M checkpoint regulators, such as CHK1/2 and WEE1, has been reported to induce synthetic lethality in cancer cells expressing mutp53. CHK1 inhibitors, such as UCN-01, PF477736, SCH900776, and AZD-7762, potentiate the cytotoxicity of topoisomerase inhibitors and ionizing radiation in cancer cells with mutp53 (Qiu et al., 2018). AZD1775, a specific WEE1 inhibitor, displays a synergistic cytotoxicity with the chemotherapeutic agent gemcitabine or the PARP1 inhibitor olaparib in p53-mutant ovarian and endometrial cancer cells (Meng et al., 2018). Furthermore, AZD1775 was shown to enhance carboplatin efficacy in p53-mutant tumors refractory or resistant (<3 months) to first-line platinum-based therapy in a phase II clinical trial (Leijen et al., 2016). However, it remains unclear whether the synthetic lethality in some of these studies is dependent on the loss of wild-type p53 function or mutp53 GOF activity. Notably, it was reported that GOF mutp53 upregulates CHK1 expression to prevent collapse of replication forks, and CHK1 inhibitor PF477736 suppresses the growth of lung cancer xenograft tumors in mice in a GOF mutp53-dependent manner (Singh et al., 2017). GOF mutp53 was also reported to enhance the expression of proteasome genes to protect cancer cells against proteotoxic stress, conferring cancer cells resistance to proteasome inhibitors (Walerych et al., 2016). Combination of PRIMA-1MET and carfilzomib, a proteasome inhibitor used as an anti-cancer drug, inhibits breast xenograft tumors in mice in a mutp53-dependent manner (Walerych et al., 2016). GOF mutp53 impairs DNA repair through interacting with PARP1 (Polotskaia et al., 2015). The PARP inhibitor talazoparib in combination with temozolomide, an alkylating agent, was reported to display synergistic cytotoxicity in breast cancer cells expressing GOF mutp53 (particularly R273H mutp53) (Xiao et al., 2020).

Some recent studies have also shown the potential application of immunotherapies for treating cancers with mutp53. The infiltration of mutp53-reactive T cells has been identified in ovarian cancer metastases (Deniger et al., 2018). The immunogenicity of mutp53 has been confirmed by a p53-specific screening assay showing that mutp53 neoantigens can be recognized by tumor-infiltrating lymphocytes in patients with epithelial cancers (Malekzadeh et al., 2019). These studies suggest that adoptive T-cell therapy is a potential therapeutic strategy to directly target mutp53.

It is worth noting that a recent study analyzing the genome sequence of 140 human embryonic stem cell lines, including 26 cell lines prepared for potential clinical use, reported that the mutation of p53 gene increases with passage number under standard culture conditions, suggesting a selective advantage of the p53 mutations (Merkle et al., 2017). Given that human pluripotent stem cells (hPSCs) have become an attractive source for regenerative therapies and the critical role of p53 in tumor suppression, careful genetic characterization of hPSCs and their derivatives should be carried out before clinical use (Merkle et al., 2017).

## Concluding remarks

Since p53 gene is mutated in >50% of all cancers and mutp53 often displays GOF activities in tumorigenesis, mutp53 has become an attractive target for cancer therapy. p53 has been discovered for over 40 years, and p53 is one of the most extensively studied proteins; however, majority of studies on p53 have been focused on the wild-type p53. Although significant progress has been made in our studies on mutp53 in cancer during the past decade, our understanding of the role and mechanism of mutp53 regulation and GOF activities in cancer is still limited, with many questions unresolved. For instance, while well established by tremendous *in vitro* cell experiments and various animal models, the mutp53 GOF needs to be further validated in clinical studies. Furthermore, majority of mutp53 GOF studies have been focused on several hotspot p53 mutants in cancer, while it remains unclear whether other nonhotspot p53 mutants can exert similar GOF activities through similar mechanisms. Based on the results from previously published studies, mutp53 GOF effects and mechanisms appear to vary in different contexts, potentially depending on mutation positions, cell and tissue types, cancer types, and even the tumor microenvironment and stress signals. This complexity presents challenges in developing some general therapeutic strategies to target different GOF mutp53 in different types of cancers. So far, different mutp53-targeted therapeutic strategies, as summarized above, have been developed, shown to be effective to certain extent and promising in preclinical studies, and some even entered clinical trials; however, there are still unresolved obstacles in mutp53-targeted cancer therapy, and currently, there are no approved drugs for clinical treatment of cancers expressing mutp53. Obviously, more studies on mutp53 regulation and GOF and mutp53-targeted therapies are necessary, which will lead to more effective and precise therapies targeting mutp53 in cancers.

## Funding

This work was supported in part by grants from the National Institutes of Health (NIH; R01CA227912 and R01CA214746) to Z.F. and grants from NIH (R01CA203965) and Congressionally Directed Medical Research Programs (CDMRP; W81XWH-16-1-0358 and W81XWH1810238) to W.H. 

**Conflict of interest:** none declared. 
